# Distinguishing Antimicrobial Models with Different Resistance Mechanisms via Population Pharmacodynamic Modeling

**DOI:** 10.1371/journal.pcbi.1004782

**Published:** 2016-03-11

**Authors:** Matthieu Jacobs, Nicolas Grégoire, William Couet, Jurgen B. Bulitta

**Affiliations:** 1 INSERM U1070, Poitiers, France; 2 Center for Pharmacometrics and Systems Pharmacology, Department of Pharmaceutics, College of Pharmacy, University of Florida, Gainesville, Florida, United States of America; University of Houston, UNITED STATES

## Abstract

Semi-mechanistic pharmacokinetic-pharmacodynamic (PK-PD) modeling is increasingly used for antimicrobial drug development and optimization of dosage regimens, but systematic simulation-estimation studies to distinguish between competing PD models are lacking. This study compared the ability of static and dynamic *in vitro* infection models to distinguish between models with different resistance mechanisms and support accurate and precise parameter estimation. Monte Carlo simulations (MCS) were performed for models with one susceptible bacterial population without (M1) or with a resting stage (M2), a one population model with adaptive resistance (M5), models with pre-existing susceptible and resistant populations without (M3) or with (M4) inter-conversion, and a model with two pre-existing populations with adaptive resistance (M6). For each model, 200 datasets of the total bacterial population were simulated over 24h using static antibiotic concentrations (256-fold concentration range) or over 48h under dynamic conditions (dosing every 12h; elimination half-life: 1h). Twelve-hundred random datasets (each containing 20 curves for static or four curves for dynamic conditions) were generated by bootstrapping. Each dataset was estimated by all six models via population PD modeling to compare bias and precision. For M1 and M3, most parameter estimates were unbiased (<10%) and had good imprecision (<30%). However, parameters for adaptive resistance and inter-conversion for M2, M4, M5 and M6 had poor bias and large imprecision under static and dynamic conditions. For datasets that only contained viable counts of the total population, common statistical criteria and diagnostic plots did not support sound identification of the true resistance mechanism. Therefore, it seems advisable to quantify resistant bacteria and characterize their MICs and resistance mechanisms to support extended simulations and translate from *in vitro* experiments to animal infection models and ultimately patients.

## Author Summary

Mathematical models are increasingly used for analysis and interpretation of in vitro efficacy results of antimicrobial drugs. Various models are employed in the scientific literature and it seems that they are equally able to describe the observed data. The aim of the present study was to compare different models in various experimental designs and with different resistance mechanisms of bacteria. For that purpose we have generated experimental data through Monte-Carlo simulations and then used six different mathematical models to analyze these results. We showed that statistical comparison of models did not allow determining which was the true mechanism of resistance, i.e. the one used for the simulation step. Moreover mathematical parameters for bacterial resistance were estimated with bias and with a low precision except for the simpler cases. This suggests that the choice of the mathematical model for data analysis should be guided by experimental characterization of the bacterial mechanism of resistance.

## Introduction

Antimicrobial therapy greatly benefits from optimized antibiotic dosage regimens that are supported by pharmacokinetic (PK) and pharmacodynamic (PD) concepts. Traditionally, the minimum inhibitory concentration (MIC) has been the predominant measure of bacterial susceptibility to predict antibiotic efficacy and it still is considered as a ‘gold standard’ for determining the bacterial susceptibility and predicting therapeutic success [1]. As most antibiotics have been available for well over a decade, their development relied heavily on MIC based approaches [2].

Despite its popularity, the MIC is subject to several limitations. It is determined at only one time point (usually between 16 and 24h), at a low initial bacterial inoculum (*i*.*e*. usually in the absence of resistant populations), and utilizes constant (*i*.*e*. static) antibiotic concentrations [1]. Therefore, the MIC neither provides information on the time-course of bacterial killing [2–4] nor on emergence of resistance [5, 6].

There are several types of mechanisms which contribute to bacterial resistance [7]. Heteroresistance defines the scenario where a small number of resistant bacteria (e.g. 0.0001% [equivalent to 10^−6^] of the initial inoculum) are present before initiation of antibiotic therapy [8]. Such a small resistant population is typically not found in standard microbroth MIC tests due to the small initial inoculum used for MIC testing. However, serious infections with a high bacterial burden almost certainly harbor such resistant bacteria at initiation of therapy [7]. Secondly, bacterial adaptation decreases the bacterial susceptibility due to the up-regulation of a resistance mechanism (such as an efflux pump in response to a quinolone antibiotic or the AmpC β-lactamase enzyme in response to β-lactam antibiotics that bind penicillin-binding protein 4 in Pseudomonas [9]). Finally, a phenotypic transition between normal replicating bacteria and tolerant bacteria with a greatly reduced growth rate can result in reduced drug sensitivity [10].

To address some of the limitations of the MIC approach, many static and dynamic *in vitro* and *in vivo* infection model studies have assessed the ability of empirical PK/PD indices to predict the efficacy of antibiotics. Such data have proven useful to optimize antibiotic monotherapy regimens for patients [2, 11, 12]. The large majority of murine infection model studies only assessed bacterial counts at one time point (usually 24 h) and did not assess the time-course of bacterial killing *in vivo*.

*In vitro* infection model experiments [13] use either static antibiotic concentrations [6, 10, 14–17], simulate the dynamic time-course of antibiotic concentrations observed in patients [5, 18–20], or utilize both of these approaches [21–23]. These experimental models provide a wealth of time-course data on bacterial growth and killing [24]. While a series of time-course models for bacterial growth, killing and emergence of resistance has been proposed, it is currently unknown which type of dataset from *in vitro* studies is required to soundly develop such PK/PD time-course models. We suspected datasets which only contain viable counts of the total bacterial population, but do not contain data on resistant population(s), may be insufficient to distinguish between competing models with different resistance mechanisms. We are also not aware of a systematic simulation-estimation study assessing the bias and precision of parameter estimates from *in vitro* antibacterial models.

Therefore, our objective was to compare the ability of static and dynamic *in vitro* infection models to identify the PK/PD model with the true resistance mechanism used during simulation and to estimate model parameters accurately and precisely. We used Monte Carlo simulations based on six candidate models and estimated these PK/PD models via importance sampling in the S-ADAPT software which is a robust and one of the latest population estimation algorithms.

## Materials and Methods

**Fig 1:**
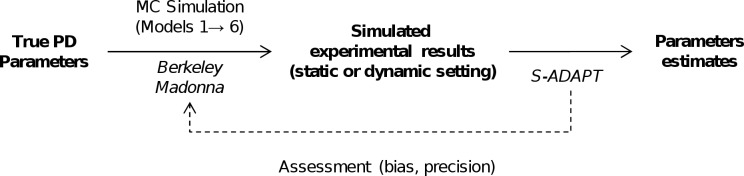
Simulation-estimation flow chart. Based on a set of true PD parameter values, Monte Carlo (MC) simulations were performed using six PD models to generate experimental datasets under both static and dynamic conditions. Each of these datasets was then estimated via population PK/PD modeling in S-ADAPT for each of the six models (yielding 36 simulation-estimation scenarios in total). The parameter estimates for each model were compared to the true parameter values used during simulation to assess bias and precision.

**Fig 2:**
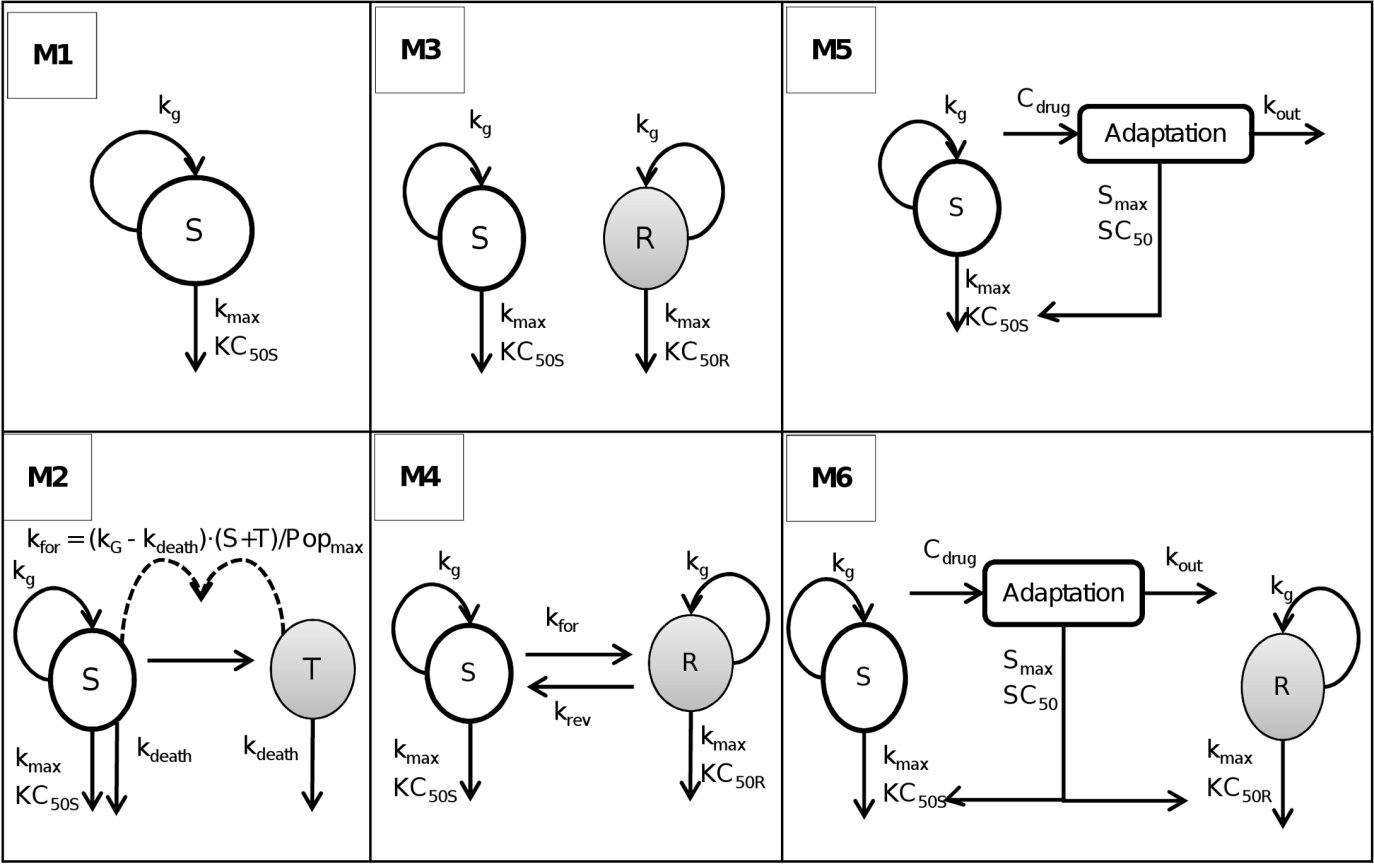
Structure of the PK/PD models used for Monte Carlo simulations and subsequent estimations. Parameters are explained in the method section and in Table 1. S, compartment with antibiotic-susceptible bacteria; T, compartment with resting, antibiotic-tolerant bacteria; R, compartment with less susceptible (resistant) bacteria.

The overview flow chart (**Fig 1**) summarizes the simulation-estimation procedures. Six different PD models were selected to reflect a range of relevant PD models for antibiotics (**Fig 2**). These models represent within-host antibiotic resistance models which seek to optimize the probability of patient cure and prevention of resistance at the individual resistance level. This should be distinguished from between-host antibiotic resistance models [25] which seek to minimize resistance by preventing transmission of resistant bacteria between patients.

The within-host resistance models evaluated in the present work contained one bacterial population with no emergence of resistance (M1), one population with the capacity to convert into a resting stage (M2), presence in the initial inoculum of two bacterial populations with different susceptibility to an antibiotic (M3 and M4) and one or two bacterial populations with a reversible adaptation to the antibiotic (M5 and M6). Models M3, M4 and M6 represent heteroresistance of the initial inoculum and models M5 and M6 incorporate adaptive resistance. The latter was described by a turnover model to describe the stimulation of adaptive resistance in response to an antibiotic and reversion back to baseline after removal of the antibiotic (**Fig 2**). Monte Carlo (MC) simulations were performed based on each of these six PD models to generate 1,200 simulated datasets for the static (20 viable count profiles per dataset) or dynamic setting (four profiles per dataset).

### Pharmacodynamic models

All six models contained a logistic growth function to limit growth to a maximum total bacterial population size (Popmax). Model 1 (M1) contained one bacterial population and bacterial killing followed an Emax model. This model was originally proposed for antibiotic PD by Zhi *et al*. [26] and subsequently used by other investigators [3, 27]. The differential equation for the number of viable, susceptible bacteria (S) was:

$$\frac{\text{dS}}{\text{dt}}=\left[\;{\text{k}}_{\text{g}}\;\cdot \;\left(1-\frac{\text{S}}{\text{Popmax}}\right)\;-\frac{\text{kmax}\;\cdot \;\text{C}}{{\text{KC}}_{50\text{S}}+\text{C}}\right]\cdot \;\text{S}$$ (1)

Initial condition (IC): S_0_ = 10^Inoc^.

The k_g_ is the apparent growth rate constant, C the antibiotic concentration, Popmax the maximum concentration of bacteria, k_max_ the maximal rate constant of bacterial killing, and KC_50S_ the antibiotic concentration yielding 50% of k_max_ for susceptible bacteria. The mean generation time (MGT) was calculated as the inverse of k_g_.

Model 2 was adapted from Nielsen *et al*. [10] (**Fig 2**). The total bacterial population is comprised of two populations (*i*.*e*. two stages), one replicating and sensitive stage and one resting and antibiotic tolerant stage. The total bacterial population is assumed to stimulate the transfer of bacteria from the sensitive stage to the tolerant stage. The transfer from the resting stage to the sensitive stage was assumed to be negligible and fixed to zero following the original publication [10]. Bacteria in the resting stage did not grow and were not killed by the antibiotic. As resting bacteria did not revert back to the proliferating stage, they were only subject to a slow first-order natural death process. Therefore, these tolerant bacteria are (slowly) dying which causes a biphasic killing profile at high antibiotic concentrations. In this model, bacteria in the tolerant stage can however not repopulate the replicating population as the reversion to the sensitive replicating stage was assumed to be zero.

As bacteria were simulated to be in the early logarithmic growth phase, we assumed that only a small fraction (10^−5^) of bacteria in the starting inoculum was in the resting, antibiotic-tolerant stage (T). This choice had very limited impact on the model, as this fraction (10^−5^) is equivalent to the number of bacteria that convert from the S to the T stage in approximately 1 min for a 10^6^ CFU/mL inoculum.

Both populations were assumed to have the same first-order natural death rate constant (k_death_). The differential equations for the sensitive (S) and resting (T) population of model 2 are:

$$\frac{\text{dS}}{\text{dt}}=\left[\;{\text{k}}_{\text{g}}\;\cdot \;\left(1-\frac{\text{S}\;+\;\text{T}}{\text{Popmax}}\right)-\frac{\text{kmax}\;\cdot \;\text{C}}{{\text{KC}}_{50}\;+\;\text{C}}-{\text{k}}_{\text{death}}-{\text{k}}_{\text{for}}\;\right]\;\cdot \;\text{S}\;\text{IC}:\;{\text{S}}_{0}=10^{\text{Inoc}}\;-\;{\text{R}}_{0}$$ (2)

$$\frac{\text{dT}}{\text{dt}}={\text{k}}_{\text{for}}\;\cdot \;\text{S}\;-\;{\text{k}}_{\text{death}}\;\cdot \;\text{T}\;\text{IC}:\;{\text{R}}_{0}=10^{\text{Inoc}\;-\;5}$$ (3)

Where ${\text{k}}_{\text{for}}=\left({\text{k}}_{\text{g}}-{\text{k}}_{\text{death}}\right)\;\cdot \;\left(\frac{\text{S}\;+\;\text{T}}{\text{Popmax}}\right)$

The k_death_ was calculated as the inverse of the mean natural death time (MDT).

Model 3 (**Fig 2**) was derived from Jumbe *et al*. [28], Gumbo *et al*. [29] and Campion *et al*. [18]. This model included a pre-existing susceptible (S) and a pre-existing resistant (R) population. Both populations did not interconvert. The initial condition of the resistant population is the mutation frequency (mutf) multiplied by the total inoculum and the initial condition of the susceptible population is the remainder of bacteria. Both populations were assumed to have the same maximal killing rate constant (k_max_) and the same growth rate constant (k_g_). These populations differed however in their drug concentration yielding 50% of kmax. The drug concentration causing 50% of kmax was smaller for the susceptible population (KC_50S_) than the drug concentration causing 50% of kmax for the resistant population (KC_50R_). This yields the following differential equations for model 3:

$$\frac{\text{dS}}{\text{dt}}=\left[\;{\text{k}}_{\text{g}}\;\cdot \;\left(1-\frac{\text{S}+\text{R}}{\text{Popmax}}\right)-\frac{\text{kmax}\;\cdot \;\text{C}}{{\text{KC}}_{50\text{S}}\;+\;\text{C}}\right]\cdot \;\text{S}\;\text{IC}:\;{\text{S}}_{0}=10^{\text{Inoc}}\cdot (1\;-\;10^{\text{mutf}})$$ (4)

$$\frac{\text{dR}}{\text{dt}}=\left[\;{\text{k}}_{\text{g}}\;\cdot \;\left(1-\frac{\text{S}\;+\;\text{R}}{\text{Popmax}}\right)-\frac{\text{kmax}\;\cdot \;\text{C}}{{\text{KC}}_{50\text{R}}\;+\;\text{C}}\right]\;\cdot \;\text{R}\;\text{IC}:\;{\text{R}}_{0}=10^{\text{Inoc}\;-\;\text{mutf}}$$ (5)

In comparison to model 3, model 4 (M4) contained an additional bi-directional inter-conversion between the susceptible and resistant population (**Fig 2**). Model 4 was derived from Jusko *et al. [30]* and Yano *et al*. [16]. The initial inoculum of the resistant population was assumed to be in equilibrium (*i*.*e*. steady-state) with the susceptible population. Therefore, the initial condition of the resistant population was calculated as CFU_0_ ∙ k_for_ / k_rev_ and the initial condition of the susceptible population was CFU_0_ ∙ (1—k_for_ / k_rev_). Model 4 was described by the following differential equations:

$$\frac{\text{dS}}{\text{dt}}=\left[\;{\text{k}}_{\text{g}}\;\cdot \;\left(1-\frac{\text{S}\;+\;\text{R}}{\text{Popmax}}\right)-\frac{\text{kmax}\;\cdot \;\text{C}}{{\text{KC}}_{50\text{S}}\;+\;\text{C}}\right]\;\cdot \;\text{S}\;-\;{\text{k}}_{\text{for}}\;\cdot \text{S}\;+\;{\text{k}}_{\text{rev}}\;\cdot \text{R}\;\text{IC}:\;{\text{S}}_{0}=10^{\text{Inoc}}\;\cdot (1\;-\;{\text{k}}_{\text{for}}\;/\;{\text{k}}_{\text{rev}})$$ (6)

$$\frac{\text{dR}}{\text{dt}}=\left[\;{\text{k}}_{\text{g}}\;\cdot \left(\text{1}-\frac{\text{S}\;+\;\text{R}}{\text{Popmax}}\right)-\frac{\text{kmax}\;\cdot \;\text{C}}{{\text{KC}}_{\text{50R}}\;+\;\text{C}}\right]\cdot \;\text{R}+{\text{k}}_{\text{for}}\;\cdot \text{S}-{\text{k}}_{\text{rev}}\;\cdot \text{R}\;\text{IC}:\;{\text{R}}_{0}=10^{\text{Inoc}}\;\cdot \;{\text{k}}_{\text{for}}\;/\;{\text{k}}_{\text{rev}}$$ (7)

The k_for_ and k_rev_ are the first-order transfer rate constants from the susceptible to the resistant population and *vice versa*.

Model 5 contained one bacterial population with adaptive resistance. An adaptive resistance model has been proposed previously by Tam *et al*. [31]. In the present study, we propose a new adaptation function that was based on an indirect response model to reflect the situation that bacteria often need to synthesize a protein (and other biomolecules) to (over-)express a resistance mechanism. The synthesis and turnover of such molecules can be captured by a turnover model. In the present model, the adaptation compartment affected the KC_50S_ to reflect the up-regulation of a bacterial efflux pump. The differential equations for model 5 were:

$$\frac{\text{dS}}{\text{dt}}=\left[\;{\text{k}}_{\text{g}}\;\cdot \left(1-\frac{\text{S}}{\text{Popmax}}\right)-\frac{\text{kmax}\;\cdot \;\text{C}}{{\text{KC}}_{50\text{S}}\;+\;\text{C}}\right]\cdot \;\text{S}\;\text{IC}:\;{\text{S}}_{0}=10^{\text{Inoc}}$$ (8)

$$\frac{\text{d}\left(\text{Adaptation}\right)}{\text{dt}}=\left(\frac{\text{Smax}\;\cdot \;\text{C}}{{\text{SC}}_{50}\;+\;\text{C}}-\text{Adaptation}\right)\cdot \;{\text{k}}_{\text{out}}\;\text{IC}:\;{\text{Adaptation}}_{0}=0$$ (9)

$${\text{KC}}_{50\text{S}}={\text{KC}}_{50,\text{base}}\;\cdot \left(1+\text{Adaptation}\right)$$ (10)

The adaptation variable defines the extent of change of KC_50S_ in response to a bacterial alteration (such as the expression of an efflux pump; e.g. MexXY-OprM in response to an aminoglycoside) [32, 33]. The KC_50,base_ is the antibiotic concentration causing 50% of kmax in absence of adaptation (*e*.*g*. at time zero), S_max_ the maximum fold-increase of KC_50S_ due to adaptive resistance, SC_50_ the drug concentration that yields 50% of S_max_, and k_out_ the first order turnover rate constant for adaptation. The k_out_ was calculated as the inverse of the mean turnover time (MTT_loss_). In contrast to an earlier model for adaptive resistance [31], the time to adaptation for the present turnover model is determined by the mean turnover time of adaptive resistance and is thus independent of the antibiotic concentration.

Models 3 and 6 both contained two pre-existing populations with different susceptibility. In contrast to model 3, model 6 contained the same adaptation function as model 5 which affected both the susceptible (KC_50S_) and the resistant (KC_50R_) population in model 6.

$$\frac{\text{dS}}{\text{dt}}=\left[\;{\text{k}}_{\text{g}}\;\cdot \left(1-\frac{\text{S}\;+\;\text{R}}{\text{Popmax}}\right)-\frac{\text{kmax}\;\cdot \;\text{C}}{{\text{KC}}_{50\text{S}}\;+\;\text{C}}\right]\cdot \;\text{S}\;\text{IC}:\;{\text{S}}_{0}=10^{\text{Inoc}}\;\cdot (1\;-\;10^{\text{mutf}})$$ (11)

$$\frac{\text{dR}}{\text{dt}}=\left[\;{\text{k}}_{\text{g}}\;\cdot \left(1-\frac{\text{S}\;+\;\text{R}}{\text{Popmax}}\right)-\frac{\text{kmax}\;\cdot \;\text{C}}{{\text{KC}}_{50\text{R}}\;+\;\text{C}}\right]\cdot \;\text{R}\;\text{IC}:\;{\text{S}}_{0}=10^{\text{Inoc}}\;\cdot 10^{\text{mutf}}$$ (12)

$$\frac{\text{d}\left(\text{Adaptation}\right)}{\text{dt}}=\left(\frac{\text{Smax}\;\cdot \;\text{C}}{{\text{SC}}_{50}+\text{C}}-\text{Adaptation}\right)\cdot {\text{k}}_{\text{out}}\;\text{IC}:\;{\text{Adaptation}}_{0}=0$$ (13)

$${\text{KC}}_{50\text{S}}={\text{KC}}_{50\text{S},\text{base}}\;\cdot \left(1+\text{Adaptation}\right)$$ (14)

$${\text{KC}}_{50\text{R}}={\text{KC}}_{50\text{R},\text{base}}\;\cdot \left(1+\text{Adaptation}\right)$$ (15)

These six PD models can be readily expanded by including different mean generation times and different maximum killing rate constants for the susceptible and resistant population. However, for the purposes of this simulation estimation study, the simpler version of these models was preferred to support parameter estimation.

### Monte Carlo simulations for static and dynamic *in vitro* infection models

#### Population mean estimates

Informed by the range of ed parameter values for different antibiotics and bacterial strains [3, 6, 14, 16, 20, 21, 23, 34–37] (**Table 1**), we selected sets of parameter values that yielded comparable CFU vs. time profiles over 24 h for simulation from the six models. These simulations were intended to provide realistic datasets on the antibiotic concentration effect-relationship for the viable counts of the total population for *in vitro* time-kill experiments. A starting inoculum of 10^6^ CFU/mL was applied for all simulations.

**Table 1:** Parameter values used for Monte Carlo simulations

Descriptions	Parameters	Units	Used in models	Mean (= true value used during simulations)
Mean generation time	**MGT**	min	1–6	60
Initial inoculum	**Inoc**	log_10_ (CFU/mL)	1–6	6
Maximum population size	**Popmax**	log_10_ (CFU/mL)	1–6	9.5
Maximum killing rate constant	**kmax**	h^-1^	1–6	4
Antibiotic concentration yielding 50% of k_max_ for the susceptible population	**KC**_**50S**_	mg/L	1–6	1
Antibiotic concentration yielding 50% of k_max_ for the resistant population	**KC**_**50R**_	mg/L	3, 4, 6	4
Mean natural death time	**MDT**	min	2	400
Mutation frequency	**Log**_**10**_ **mutf**		3, 4, 6	-5
First-order transfer rate constant from susceptible to resistant population	**k**_**for**_	log_10_ (1/h)	4	-6
First-order transfer rate constant from resistant to susceptible population	**k**_**rev**_	log_10_ (1/h)	4	-1
Maximum fold-increase of KC_50_ due to adaptive resistance	**Smax**		5, 6	4
Antibiotic concentration that yields 50% of S_max_	**SC**_**50**_	mg/L	5, 6	0.4
Mean turnover time for adaptive resistance	**MTT**_**loss**_	h	5, 6	20

^a^: All parameters were simulated with a small between curve variability to represent generally well reproducible *in vitro* curves. Parameter were assumed to follow a log-normal distribution and were simulated with a 10% coefficient of variation for the between curve variability. Parameters estimated on log_10_ scale (see unit column) were simulated via a normal distribution on log_10_ scale and had a standard deviation of 0.05.

#### Between curve variability and residual error

For these well-controlled *in vitro* studies, between curve variability was set to a small coefficient of variation of 10% for log-normally distributed parameters and to a standard deviation of 0.05 on log_10_ scale for normally distributed parameters. All models were simulated and estimated using a major-diagonal variance-covariance matrix. The residual error of log_10_ CFU/mL counts was additive with a standard deviation of 0.2 on log_10_ scale. A lower limit of quantification (LLOQ) of 10 CFU/mL was chosen [21] (equivalent to one colony per agar plate for a volume of 100 uL bacterial suspension per agar plate).

#### Simulated experimental designs

The experimental designs used for simulation are summarized in **Table 2**. To mimic typical experimental conditions, ten different constant antibiotic concentrations were simulated for each static time-kill experiment. These concentrations were 0 mg/L (control), and 0.125, 0.25, 0.5, 1, 2, 4, 8, 16, and 32 times KC_50S_ (assumed to be 1 mg/L). Each concentration was simulated in duplicate yielding 20 curves per static time-kill dataset. Viable counts of the total population were observed at 0 (pre-dose), 0.5, 1, 2, 4, 8, 12 and 24 h.

**Table 2:** Conditions used for Monte Carlo simulations of static and dynamic *in vitro* infection models.

Experi-mental condition	Antibiotic concentration (x KC_50S_)	Sampling times (h)	Simulated elimination half-life (h)	Dosing interval (h)	Number of models used for simulation	Number of experiments simulated for each model
Static	0, 0.125, 0.25, 0.5, 1, 2, 4, 8, 16, 32	0, 0.5, 1, 2, 4, 8, 12 and 24	(static concentration)		6	100 ^a^
Dynamic	8 (initial concentration)	0, 1, 2, 4, 8, 12, 24, 28, 32, 36 and 48	1	12h	6	100 ^b^

^a^: Each dataset for a static time-kill model contained 20 viable count profiles (including that of the growth control).

^b^: Each dataset for a dynamic infection model study contained four viable count profiles.

For dynamic one-compartment *in vitro* models, we simulated one dose level with dosing every 12 h and a growth control both in duplicate. The simulated antibiotic peak concentration was 8x KC_50S_ and the antibiotic concentrations decreased with a pharmacokinetic elimination half-life of 1 h. Bacterial counts were simulated at 0, 1, 2, 4, 8, 12, 24, 28, 32, 36 and 48 h. A short half-life has been used to allow antibiotic concentrations to change over a large range with peak concentrations yielding rapid killing and trough concentrations allowing bacterial regrowth. This choice was expected to support estimation of model parameters and emphasize the features of a dynamic infection model.

#### Monte Carlo simulations

Our simulation-estimation studies (**Fig 1**) included the generation of 200 datasets for each of the six candidate models via Monte Carlo simulations for static or dynamic antibiotic concentration-time profiles. This included 100 datasets simulated under static and another 100 datasets simulated under dynamic conditions. Each dataset comprised 20 viable count profiles of the total bacterial population for static antibiotic concentration experiments and four profiles for 1-compartment dynamic infection models. These Monte Carlo simulations were performed for six candidate models yielding 1,200 datasets in total. Berkeley Madonna (version 8.3.18, University of California) was used for all Monte Carlo simulations.

#### Estimation of population PD parameters

Each simulated dataset was estimated via population PK/PD modeling using the true model as well as the five other candidate models yielding 6 models x 1,200 datasets = 7,200 population estimations in total. All PD model parameters of the respective models were estimated by simultaneously fitting of all viable count profiles of the respective dataset (**Table 2**). Estimation was performed using nonlinear mixed-effects modeling in the S-ADAPT software via the importance sampling algorithm (pmethod = 4 in S-ADAPT) [38]. Modeling was facilitated by the SADAPT-TRAN tool and utilized estimation settings that were previously qualified for robust estimation of mechanism-based models [39, 40]. Viable counts were fitted on log_10_ scale and viable counts below the limit of counting were handled by using Beal M3 method as implemented in S-ADAPT [41].

#### PD model selection

Many of combinations of the six studied models are nested. The more complex models converge to the simpler models, if the mutation frequency of the resistant population is zero, if the maximum extent of adaptation (Smax) is zero, if there is no conversion of bacteria to a resting stage, or if there is no inter-conversion between the susceptible and resistant population. The objective function value (OFV, -1x log-likelihood in S-ADAPT) was calculated by S-ADAPT for each of the 7,200 estimation runs. For comparison of two nested models, the likelihood ratio test (LRT) with a chi-square distribution and one degree of freedom per additional model parameter was used. For comparison of two non-nested models, we chose the model with the lower objective function as the better model.

#### Precision and bias of parameters estimates

The parameter estimates from each of the estimation runs were compared with true parameter values used during simulation from the true model. The bias was calculated as.

$$Bias=\frac{Estimate\;-\;true\;value}{true\;value}$$ (16)

#### Simulation of viable count profiles

To visualize the differences between competing models for the chosen experimental conditions, we used the median parameter values (from 100 replicates per model and condition) estimated under static or dynamic conditions to simulate viable count profiles over 96 h under dynamic conditions for each model. These simulations were compared to the viable count profiles simulated based on the true parameter estimates. This simulation was meant to illustrate the impact of potentially biased parameter estimates on the predicted viable count profiles of the total population.

## Results

**Fig 3:**
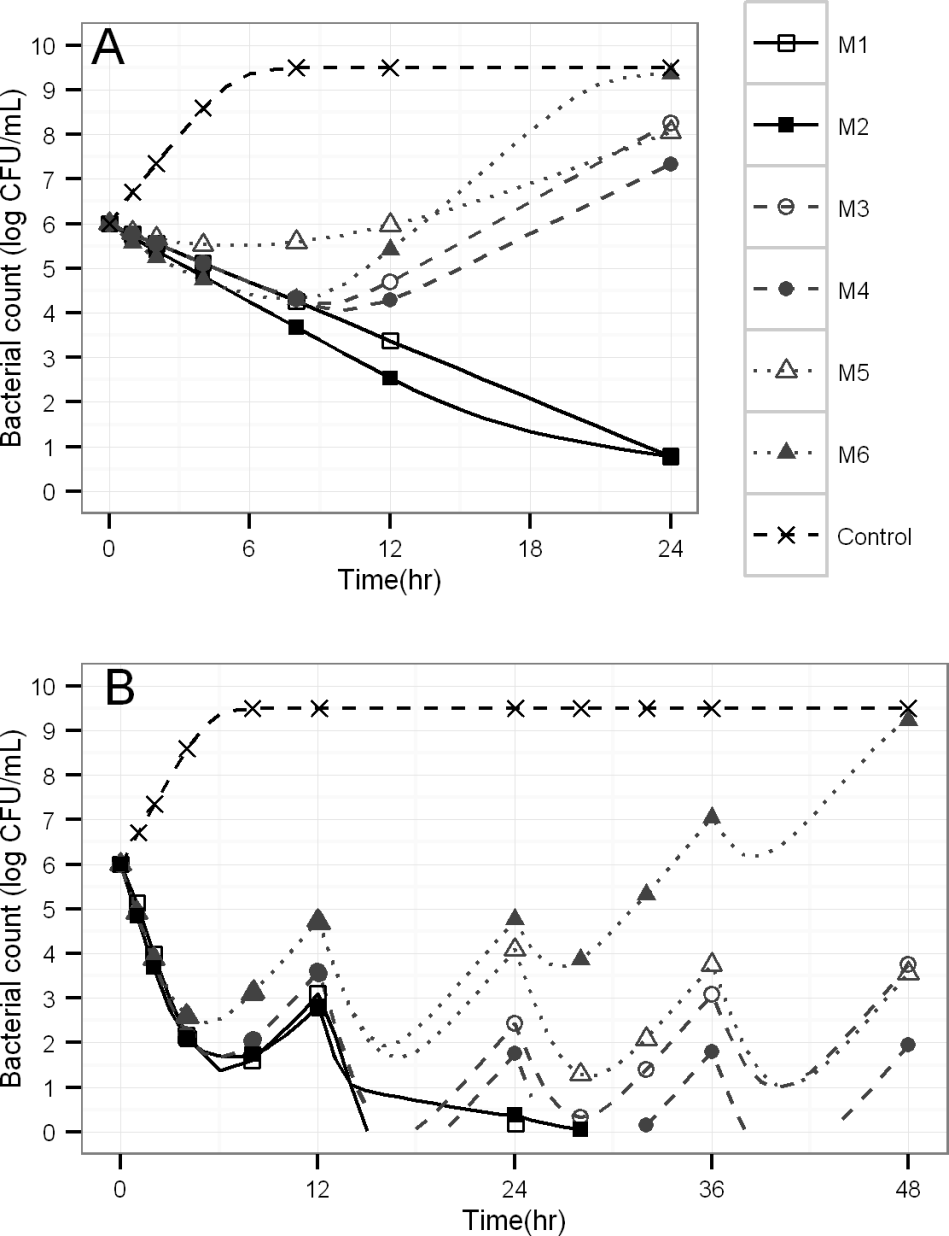
Typical viable count profiles. Simulations via six different structural models for a constant antibiotic concentration of 1 mg/L (panel A) or for multiple dosing of a hypothetical antibiotic with 1 h elimination half-life and peak concentrations of 8 times KC_50S_ (panel B).

The simulated viable counts profiles for static time-kill experiments yielded two general shapes of profiles (**Fig 3A**). The first type showed bacterial killing without regrowth (M1 and M2) with model 2 containing a slower terminal phase representing natural death of bacteria in the resting stage. The second group of profiles yielded initial bacterial killing followed by regrowth due to a resistant bacterial population (M3 and M4), adaptation (M5), or both (M6). **Fig 3B** shows the viable count profiles simulated under dynamic conditions where regrowth is in part due to low antibiotic concentrations towards the end of the 12-h dosing intervals.

**Model selection.** Each column in Table 3 refers to one true model used for simulation under static or dynamic conditions. The lines in Table 3 show the frequency of selecting the respective model as the best model. If M1 was the true model, both static and dynamic conditions identified M1 as the true model in 93% or 89% of the cases. For the model with one population with a resting stage (M2), model 2 was correctly identified as the best model in 96% of cases for the static scenario but only in 8% of the cases for the dynamic scenario. When model 3 was used as true model for simulations, static conditions correctly identified M3 as the best model in 82% and dynamic conditions in 97% of the cases (**Table 3**).

**Table 3:** Probability of selecting a model (M1 to M6) as the best model (lines) for six different true models (columns) used for simulation under dynamic or static conditions. The probability to correctly select the true model as the best model is represented by the diagonal (bold numbers).

		Actual (*i*.*e*. true) model											
Condition Models		Static time-kill						Dynamic infection model					
		M1	M2	M3	M4	M5	M6	M1	M2	M3	M4	M5	M6
Selected model (%)	M1	**93**	.	.	.	.	.	**89**	87	.	.	.	.
	M2	7	**96**	.	.	.	.	3	**8**	.	.	.	.
	M3	.	3	**82**	81	84 ^a^	44	6	3	**97**	96	84 ^a^	90
	M4	.	.	17	**18**	2	2	.	1	.	**1**	2	.
	M5	.	.	.	.	**11**	.	2	1	.	1	**13**	.
	M6	.	1	1	1	3	**54**	.	.	3	2	1	**10**

^**a**^: As an example, this result means that model M3 was selected in 84% of the cases when model M5 was used as the true model during simulations both for the static and dynamic settings.

Interestingly, when the model with two populations and a slow inter-conversion (M4) was the true model used for simulations, M3 was incorrectly selected as the best model in 81% (static) or 96% (dynamic setting) of the cases. Identification of both models with adaptive resistance (M5 and M6) as the true model was only achieved in 10% to 54% of the cases under both scenarios (**Table 3**).

**Fig 4:**
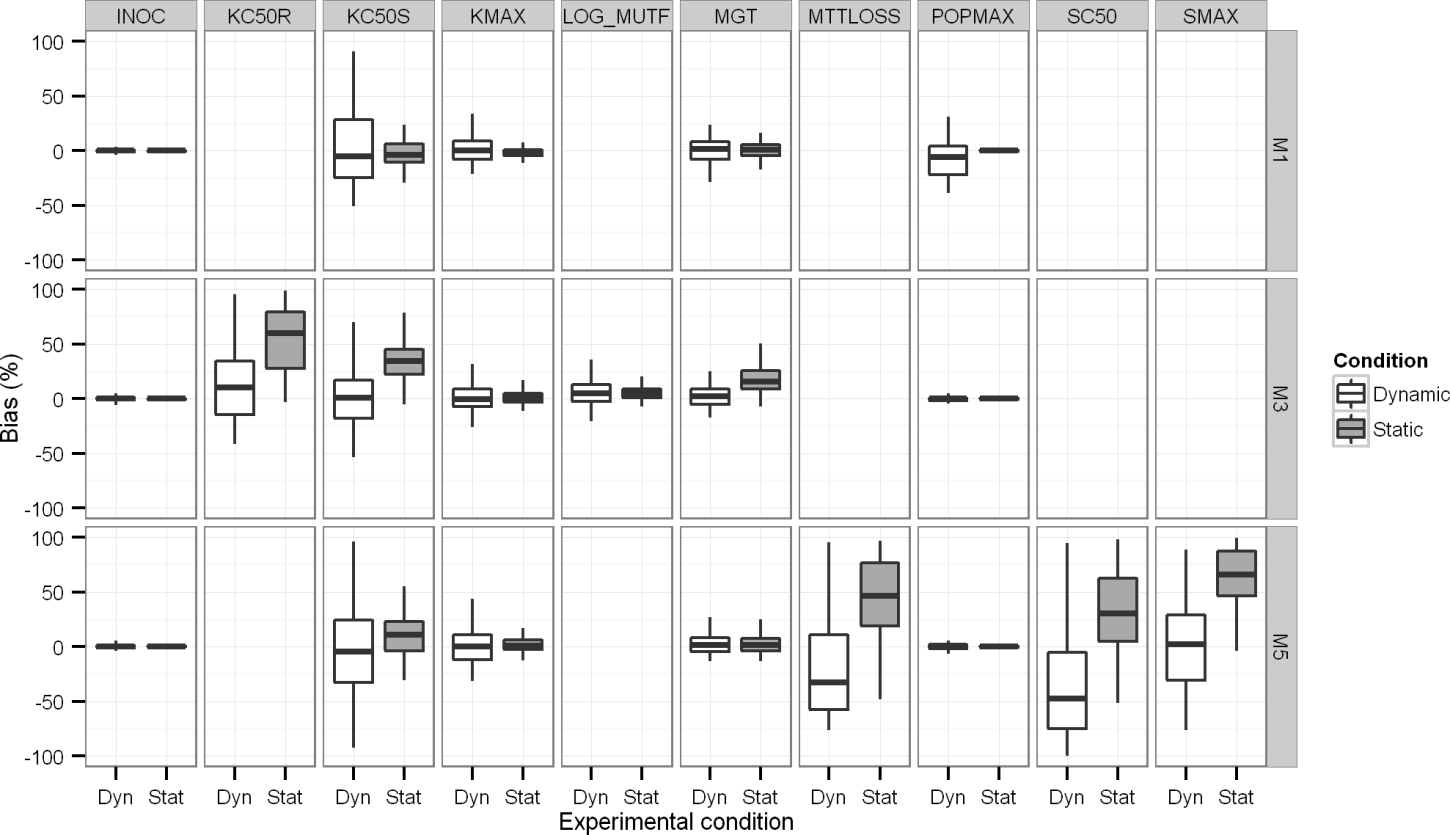
Boxplots of the bias between true and estimated parameter values under static and dynamic conditions.

**Bias and imprecision of parameter estimates. Table 4** (all six models) and **Fig 4** (models 1, 3 and 5) compare the true parameter values with the median parameter estimates under static and dynamic conditions (based on the 100 datasets for each model and case). Overall, the precision of parameter estimates tended to be better for static compared to dynamic conditions.

**Table 4:** Median and coefficient of variation (CV) of parameter estimates (n = 1200; i.e. 100 replicates for each setting and each model) under static or dynamic condition.

			Model 1		Model 2		Model 3		Model 4		Model 5		Model 6	
Parameter	True value	Unit	Static	Dynamic	Static	Dynamic	Static	Dynamic	Static	Dynamic	Static	Dynamic	Static	Dynamic
MGT	60	min	60.57 (8)	60.97 (10.7)	60.49 (8.9)	68.46 (20)	69.41 (13)	61.23 (9.0)	73.03 (11.8)	60.86 (9.7)	60.97 (12.0)	60.66 (9.9)	61.52 (6.0)	62.22 (11.8)
Inoc	6	Log_10_ CFU/mL	6.00 (0.48)	6.00 (1.8)	6.00 (0.4)	6.01 (1.6)	6.00 (0.4)	5.99 (2.3)	6.01 (0.5)	6.02 (2.0)	6.01 (0.6)	5.99 (2.0)	6.00 (0.6)	5.99 (2.3)
Popmax	9.5	Log_10_ CFU/mL	9.53 (1)	8.96 (19.8)	9.52 (1.09)	10.9 (15.9)	9.50 (0.87)	9.49 (4.4)	9.49 (0.9)	9.54 (10)	9.51 (0.7)	9.51 (8.8)	9.51 (0.76)	9.51 (1.3)
kmax	4	h^-1^	3.94 (4.2)	4.00 (11.3)	4.02 (7.8)	4.09 (15.3)	4.03 (5.7)	3.98 (12.6)	4.00 (5.0)	4.20 (12.1)	4.03 (5.6)	4.01 (15.9)	3.98 (4.4)	4.11 (18.5)
KC_50S_	1	mg/L	0.95 (14.45)	0.97 (35)	0.98 (15.6)	1.03 (40.8)	1.34 (14.0)	1.01 (38.7)	1.33 (17)	1.12 (17)	1.10 (20.0)	0.98 (59.0)	1.13 (16.9)	1.15 (59)
MDT	400	min			320.54 (18)	1279 (176.8)								
Log_10_ mutf	-5				-5.77 (13.6)	-6.36 (16.3)	-5.24 (6.0)	-5.25 (17.2)	-5.22 (9.4)	-4.89 (19.5)			-5.20 (8.0)	-5.81 (25)
KC_50R_	4	mg/L					7.26 (40.0)	4.54 (36.0)	6.35 (41.6)	3.56 (73.0)			5.24 (27.7)	6.12 (346)
k_for_	-6	Log_10_ (1/h)							-7.63 (7.9)	-7.11 (5.1)				
k_rev_	-1	Log_10_ (1/h)							< -10 (451)	-4.3 (309)				
Smax	4										9.60 (29.0)	5.68 (107.0)	5.88 (44)	6.95 (191)
SC_50_	0.4	mg/L									0.86 (51.6)	0.25 (123.2)	0.51 (106)	0.28 (199)
MTT_loss_	20	h									46.62 (37.7)	35.43 (126.3)	40.47 (33)	81.83 (92)
														

The median estimates were within 10% of the true value and the imprecision was <20% CV for most parameters of M1 and M2 under both static and dynamic conditions (**Table 4**). A noticeable exception was the estimated mean time of natural death (MDT) of resting bacteria in M2 which was considerably biased by 326% (estimated: 1,279 min *vs*. true: 400 min) in the dynamic setting and biased by 20% (estimated: 321 min *vs*. true: 400 min) in the static setting.

For the model with a susceptible and resistant population without inter-conversion (M3), the vast majority of median estimates were within 10% of the true value with exception of KC_50S_ (estimated 34% higher) and KC_50R_ (estimated 82% higher than the true value) in the static setting. Both for models 3 and 4, the dynamic setting provided less biased parameter estimates. However, the slow inter-conversion rate constants (k_for_ and k_rev_) of M4 were difficult to estimate under both settings.

For models with adaptive resistance (M5 and M6), most model parameters were estimated close to their true values and with reasonable precision. However, the parameters related to the adaptation process (*i*.*e*. Smax, SC_50_ and MTT_loss_) were considerably biased and estimated with poor precision for models M5 and M6 under both scenarios. The estimates for Smax and SC_50_ may be considered reasonable, since the mean turnover time for adaptive resistance was chosen to be 20 h and therefore almost as long as the experimental duration of 24 h for the simulated static time-kill studies. Thus, precise estimation of Smax, SC_50_ and MTT_loss_ was not expected for the chosen parameter values and experimental design.

**Fig 5:**
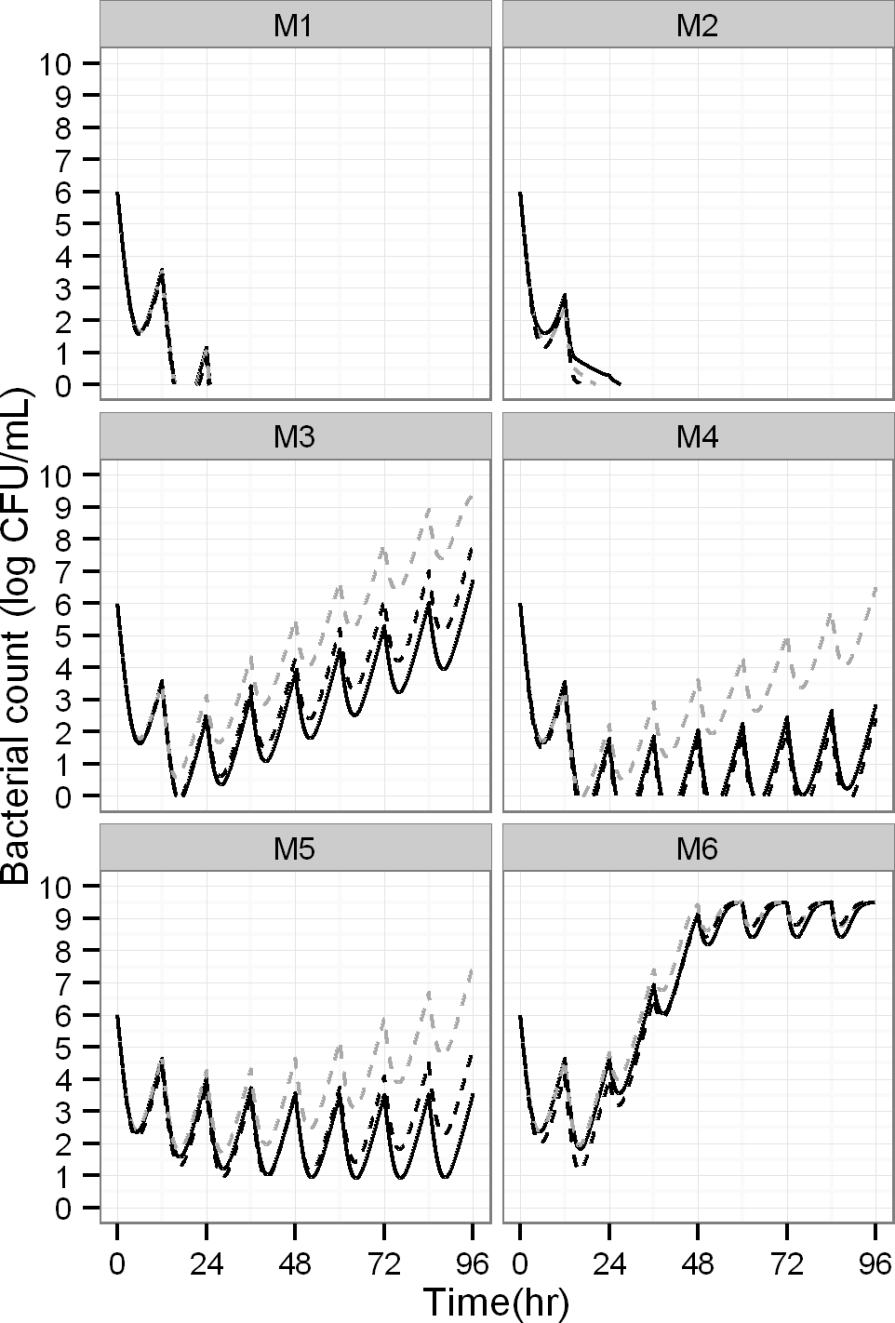
Simulated viable count profiles after multiple dosing under dynamic conditions for a hypothetical antibiotic with a 1 h elimination half-life and peak concentration of 8 x KC_50S_. Simulations were based on the true parameter values (solid black lines) or parameter values estimated under dynamic (dashed black) or static conditions (dashed grey).

**Impact of biased parameter estimates on viable counts.** The viable count profiles predicted from the median estimates under static and dynamic conditions (**Fig 5**) matched the predicted profiles from the true parameter estimates closely during the first 48 h. For models 3, 4 and 5, the deviations were moderate between 48 and 96 h and tended to be larger for the model predictions under static compared to dynamic conditions (**Fig 5**). Predictions were slightly better for the two population model without adaptation (M3) than those for the model containing one population with adaptation (M5). Although some of the parameter estimates were biased for the more complex model M6 (two populations with adaptation), the predictions matched the observations over 96 h closely (**Fig 5**).

## Discussion

During the last five decades, a considerable variety of structures for models with irreversible drug effects has been proposed in antimicrobial and antineoplastic chemotherapy [3, 7, 42]. These published models include both empiric descriptions of viable count profiles and mechanism-based models. The latter models were developed to characterize relevant aspects of the mechanisms of antibiotic action, bacterial resistance and tolerance for antibiotic mono- and combination therapy and are highly useful to predict the time-course of bacterial growth, killing and resistance and to thereby optimize antibiotic dosage regimens. The vast majority of these antibacterial PK/PD models [3, 7, 42] were developed using data on the total bacterial population and did not model viable counts from antibiotic containing agar plates. However, several models co-modelled both the total and resistant populations [28, 43, 44].

In this context, it seems surprising that no systematic simulation-estimation study has yet been published to assess the ability to distinguish between competing antimicrobial PD models with different resistance mechanisms. This lack of knowledge affects the vast majority of mathematical models in antibacterial PD. We addressed this gap by performing Monte Carlo simulations with in total 1,200 simulated datasets that were estimated using six relevant structural models and two common designs for *in vitro* infection models. These models reflected genotypically stable resistance mechanisms (model M3), phenotypic resistance (*i*.*e*. adaptation for M5 and persisters for M2), inter-conversion between bacterial populations (M4), or multiple of these mechanisms (M6, **Fig 2**). The 1,200 datasets were estimated for both the true model and the other five models (*i*.*e*. 6 models x 1,200 datasets = 7,200 population estimations in total) to assess the ability to distinguish between competing models.

While models M1 and M2 yielded bacterial killing and death without regrowth (**Fig 3A**), models M3 to M6 could all describe viable count profiles with initial killing followed by regrowth due to emergence of resistance in the presence of constant antibiotic concentrations. It was therefore interesting to assess, whether a robust population PK/PD estimation algorithm (*i*.*e*. importance sampling) could adequately distinguish between competing models. Despite the use of one of the latest population modeling algorithms, this simulation-estimation study showed that standard statistical criteria could only identify the true model under both static and dynamic conditions in more than 80% of the cases for models M1 and M3 (**Table 3**). Importantly, M3 was incorrectly selected as the best model in the large majority of cases even if models M4, M5 or M6 were the true model used during simulation. For datasets that only contained viable count profiles of the total population, statistical modeling criteria could therefore not reliably identify the true model in case of regrowth due to bacterial resistance.

To provide recommendations for the design of future experiments, the MIC can be related to the KC_50S_ as shown previously [24]. The expected KC_50S_ can then be used to guide the concentrations range to be evaluated in *in vitro* time-kill studies. An *a priori* choice would be to assess antibiotic concentrations below and above the KC_50S_ (for instance 2-fold dilutions from 0.125 to 32 times the KC_50S_). Determining the MIC at the end of the study (*e*.*g*. 24 h) experimentally yields valuable information about the extent of resistance development. This information could be subsequently used in additional experiments to assess higher antibiotic concentrations. Additionally, the level of resistance at the end of the experiment will also inform the mechanism(s) of resistance and therefore support the choice of the PK/PD model.

Quantitative viable count data of the resistant population(s) from antibiotic-containing agar plates at 0 and 24 h, for example, can provide experimental evidence to accept or reject several candidate models (**Fig 2**). If one observes resistant bacteria on antibiotic-containing agar plates (containing *e*.*g*. 3x the MIC of the antibiotic) at 0 h, one can reject models M1, M2, and M5, as those models assume the absence of resistant bacteria at time zero. Mutation frequency studies at a high bacterial density are expected to support calculation of the likelihood of pre-existing resistant mutants [7]. Quantifying and modeling resistant bacteria over time would further enhance the ability to distinguish between competing models [28, 43, 44]. An in-depth analysis of datasets containing one or multiple observations for resistant bacteria is beyond the scope of the present work. While this is a potential limitation of this simulation-estimation study, experimental data on the presence or absence of pre-existing resistant bacteria facilitated PK/PD model selection in previous studies [28, 43, 44]. The difficulty to select the most appropriate mechanism of resistance based on modelling methods alone is also supported by experience from our previous study on *P*. *aeruginosa* exposed to static concentrations of ciprofloxacin [6]. In this study, we leveraged insights on the presumably most relevant mechanism of resistance to select the final model for ciprofloxacin.

Despite considerable bias for some parameter estimates, the discrepancies between predicted and actual viable count profiles (**Fig 5**) were limited and may possibly be considered acceptable. This applies particularly for the small discrepancies during the first 24 h to 48 h which is likely the most critical time in the management of infections in critically-ill patients. Model predictions over longer time periods (*i*.*e*. extrapolation) led to more biased predictions, as expected. Our predictions were based on the median of the parameters values. As these simulations did not account for parameter imprecision, the discrepancies between the predicted and the actual viable count profiles are likely larger for some sets of estimated parameters.

Bias tended to be less for some parameters under the dynamic compared to the static experimental setting for models with heteroresistance or adaptation (M3, M4, M5 and M6; **Table 4** and **Fig 4**). As expected, the dynamic setting yielded less precise parameter estimates most likely due to the considerably smaller number of observations for the dynamic setting (containing 4 curves per dataset) compared to the static setting (containing 20 curves per dataset). In practice the statistical gain of the dynamic design can also be offset by the significantly increased workload for dynamic experimental conditions.

Static concentration time-kill studies [13] are efficient and cost-effective and allow studying a large range of antibiotic concentrations. While 24 h static time-kill studies represent the most common experimental duration, longer experimental durations could have allowed us to increase the likelihood of mathematically identifying the model with the true resistance mechanisms and to better predict antimicrobial efficacy over longer periods of time. Most published studies did not exchange the broth medium regularly (e.g. every 24 h) and therefore toxic bacterial metabolites may accumulate and nutrients get depleted over time. Also, degradation (e.g. of β-lactam antibiotics) over longer experimental conditions would need to be accounted for experimentally as we published previously [33]. Overall, performing static concentration time-kill studies over more than 24 h is fully feasible, but requires an increased amount of work.

Dynamic *in vitro* infection models such as the one-compartment and hollow-fiber system can mimic human PK [4], by changing drug concentrations and turnover of fresh broth medium using various pumps. The control of these flow rates permits to simulate different half-lives for one or multiple drugs and also provides washout of toxic bacterial metabolites. Therefore, these dynamic experiments are often run over multiple days to week and longer [31, 45] and typically use multiple dosing [46]. These dynamic *in vitro* models require a significantly enhanced workload and therefore complement and extend static concentration time-kill studies for translation to animal studies and ultimately to patients.

Some limitations of our study came from the necessity to select PD parameters characterizing the simulated pathogen, to choose an experimental design for static and dynamic kill-curves and to set the initial values for parameter estimations. Our chosen parameter values adequately characterized the concentration-effect relationship for each studied PD model and were selected based on biological plausibility and our experience from experimental datasets. Despite a clear concentration-effect relationship in the simulated viable count profiles for each dataset, it was difficult to impossible to mathematically identify the true resistance mechanism based on viable counts of the total population. It is possible that other sets of parameter estimates used during simulation would have yielded a higher likelihood to identify the model with the true resistance mechanism. This presents a potential limitation of the present study.

Moreover, the present study contained simulation-estimation scenarios for static and dynamic *in vitro* infection models. Our simulated dynamic *in vitro* model only assessed a scenario with one half-life, one dose level, and one dosing interval. Dynamic infection models with multiple doses, different dosing intervals, and potentially a range of relevant half-lives would provide a more informative dataset which may have supported the identification of the PD model with the true resistance mechanism. While it is a potential limitation of our study that we did not evaluate more dynamic infection model studies, most current papers on antimicrobial PD models use static time-kill experiments to define the concentration-effect relationship and select the PD model and its resistance mechanism. Finally, we did not assess the sensitivity of the final parameter estimates and the model selection towards the choice of the initial estimates. In our previous work, we found the importance sampling algorithm in S-ADAPT to be robust and efficient despite the use of poor (*i*.*e*. 10-fold too high or 10-fold too low) initial estimates for every structural model parameter in a complex PK/PD model [7].

In summary, for datasets based only on the total bacterial population, standard statistical modeling criteria failed to correctly identify the PD model with the true resistance mechanism(s) in the large majority of cases. These datasets did not contain data on antibiotic-resistant bacterial populations. This finding is highly important, as most published models in antibacterial PD were developed based only on data on the total bacterial population. For our simulation scenarios, dynamic infection models tended to provide more accurate parameter estimates than static concentration time-kill studies for some parameters. Static time-kill studies yielded more precise parameter estimates compared to dynamic models likely due to the larger number of profiles per datasets. For both static and dynamic conditions, parameters related to adaptive resistance and interconversion of bacterial populations tended to be poorly estimated. Interestingly, predicted viable count profiles over the experimentally studied duration (*i*.*e*. 24 to 48 h) were reasonably accurate despite biased parameter estimates. Simulations over longer durations (*i*.*e*. extrapolations) tended to show more pronounced mispredictions and should be interpreted conservatively. Overall, it seems highly beneficial to utilize quantitative viable count data of resistant populations and characterize their MICs and resistance mechanisms to support the choice of the most appropriate PD model for bacterial resistance.

## Supporting Information

S1 Table Nested models. (DOCX)

S1 Models Script for simulation with model 1. (MMD)

S2 Models Script for simulation with model 2. (MMD)

S3 Models Script for simulation with model 3. (MMD)

S4 Models Script for simulation with model 4. (MMD)

S5 Models Script for simulation with model 5. (MMD)

S6 Models Script for simulation with model 6. (MMD)

S7 Models Script for estimation with model 1. (CTL)

S8 Models Parameter settings for estimation with model 1. (CSV)

S9 Models Script for estimation with model 2. (CTL)

S10 Models Parameter settings for estimation with model 2. (CSV)

S11 Models Script for estimation with model 3. (CTL)

S12 Models Parameter settings for estimation with model 3. (CSV)

S13 Models Script for estimation with model 4. (CTL)

S14 Models Parameter settings for estimation with model 4. (CSV)

S15 Models Script for estimation with model 5. (CTL)

S16 Models Parameter settings for estimation with model 5. (CSV)

S17 Models Script for estimation with model 6. (CTL)

S18 Models Parameter settings for estimation with model 6. (CSV)

S1 Data Results for estimation in static conditions. (XLS)

S2 Data Results for estimation in dynamic conditions. (XLS)
