# Supplementary material for: Distinguishing Antimicrobial Models with Different Resistance Mechanisms via Population Pharmacodynamic Modeling
Source: PLoS Comput Biol. 2016 Mar 11;12(3):e1004782. doi: 10.1371/journal.pcbi.1004782 (PMC4788427; doi:10.1371/journal.pcbi.1004782)
Supplement: S1 Table — (DOCX) [file pcbi.1004782.s001.docx]

**Supplemental table: Nested models**

|  | Nested within model | | | | | |
| --- | --- | --- | --- | --- | --- | --- |
| Model | M1 | M2 | M3 | M4 | M5 | M6 |
| M2 | No ^a^ | n/a | No | No | No | No |
| M3 | Yes ^b^ | No | n/a |  |  |  |
| M4 | Yes ^c^ | No | Yes ^d^ | n/a |  |  |
| M5 | Yes ^e^ | No | No | No | n/a |  |
| M6 | Yes ^f^ | No | Yes ^g^ | No | Yes ^h^ | n/a |

^a^: Model M2 is not nested with any of the other models, since the conversion of bacteria from the susceptible (S) to the tolerant (T) population occurs also for small values of S+T.

^b^: Model M3 is nested within model M1, if the mutation frequency of the resistant population (R) is sufficiently small that resistant bacteria are not part of the initial inoculum (or are not observable over the tested experimental duration).

^c^: Model M4 is nested within model M1, if the mutation frequency of the resistant population (R) is sufficiently small that resistant bacteria are not part of the initial inoculum and if the conversion (k_for_) from S to R is zero.

^d^: Model M4 is nested within model M3, if the conversion (k_for_) from susceptible (S) to resistant (R) bacteria is zero (or not observable over the tested experimental duration).

^e^: Model M5 is nested within model M1, if the extent of adaptive resistance (i.e. Smax, the maximum fold-increase of KC50) is zero. The impact of adaptive resistance over the studied experimental duration will also become negligible, if the mean turnover time for adaptive resistance (MTTloss = 1/k_out_) becomes very long (e.g. >100 h).

^f^: Model M6 is nested within model M1, if the extent of adaptive resistance is zero (i.e. Smax=0) and if the resistant population (R) is lacking.

^g^: Model M6 is nested within model M3, if the extent of adaptive resistance is zero (i.e. Smax=0).

^h^: Model M6 is nested within model M5, if the resistant population (R) is lacking.
